# *De novo* Assembly of the Indo-Pacific Humpback Dolphin Leucocyte Transcriptome to Identify Putative Genes Involved in the Aquatic Adaptation and Immune Response

**DOI:** 10.1371/journal.pone.0072417

**Published:** 2013-08-28

**Authors:** Duan Gui, Kuntong Jia, Jia Xia, Lili Yang, Jialin Chen, Yuping Wu, Meisheng Yi

**Affiliations:** 1 School of Life Sciences, Sun Yat-sen University, Guangzhou, P. R. China; 2 School of Marine Sciences, Sun Yat-sen University, Guangzhou, P. R. China; 3 Guang Dong Pearl River Estuary Chinese White Dolphin National Nature Reserve, Zhuhai, P. R. China; Auburn University, United States of America

## Abstract

**Background:**

The Indo-Pacific humpback dolphin (*Sousa chinensis*), a marine mammal species inhabited in the waters of Southeast Asia, South Africa and Australia, has attracted much attention because of the dramatic decline in population size in the past decades, which raises the concern of extinction. So far, this species is poorly characterized at molecular level due to little sequence information available in public databases. Recent advances in large-scale RNA sequencing provide an efficient approach to generate abundant sequences for functional genomic analyses in the species with un-sequenced genomes.

**Principal Findings:**

We performed a *de novo* assembly of the Indo-Pacific humpback dolphin leucocyte transcriptome by Illumina sequencing. 108,751 high quality sequences from 47,840,388 paired-end reads were generated, and 48,868 and 46,587 unigenes were functionally annotated by BLAST search against the NCBI non-redundant and Swiss-Prot protein databases (E-value<10^−5^), respectively. In total, 16,467 unigenes were clustered into 25 functional categories by searching against the COG database, and BLAST2GO search assigned 37,976 unigenes to 61 GO terms. In addition, 36,345 unigenes were grouped into 258 KEGG pathways. We also identified 9,906 simple sequence repeats and 3,681 putative single nucleotide polymorphisms as potential molecular markers in our assembled sequences. A large number of unigenes were predicted to be involved in immune response, and many genes were predicted to be relevant to adaptive evolution and cetacean-specific traits.

**Conclusion:**

This study represented the first transcriptome analysis of the Indo-Pacific humpback dolphin, an endangered species. The *de novo* transcriptome analysis of the unique transcripts will provide valuable sequence information for discovery of new genes, characterization of gene expression, investigation of various pathways and adaptive evolution, as well as identification of genetic markers.

## Introduction

The Indo-Pacific humpback dolphin, also called the Chinese white dolphin, has once widely distributed in estuarine and inshore waters of the Indian and Western Pacific Ocean [1]. Unfortunately, it has become an endangered species, and has been listed in the First Order of the National Key Protected Wild Aquatic Animals List in China and the Convention on International Trade in Endangered Species of Wild Fauna and Flora (CITES). It has been further classified on the International Union for Conservation of Nature and Natural Resources (IUCN) Red List of Threatened Species since 2012. Due to the limited available genomic information, the researches on the Indo-Pacific humpback dolphin are mainly focused on the morphology, population distribution, age structure, biodiversity, heavy metals and organic toxicants [2]–[5], and the investigations on the population genetics and evolution are still essentially rare. The Indo-Pacific humpback dolphin is particularly vulnerable to threats, such as pathogenic microorganism, persistent organic pollutants, agricultural and environmental pollutants [6]–[9]. The increasing disease susceptibility has led to a possible negative influence on the immune system and the health of this dolphin species. Because of the lack of knowledge about the cetacean immune system, the immunology of marine mammals marched slowly in the past decades. Cetaceans had undergone a radical transformation in morphology and physiology to adapt a fully aquatic lifestyle [10]. However, the molecular correlates of the remarkable phenotypic features of these aquatic mammals still remain poorly explored. The effective protection of cetacean population needs comprehensive understanding of the genetic background of the animal populations. Currently, advances in molecular techniques have enabled the study of kinship relations, genetic diversity and population structure in many different contexts, such as mtDNA (mitochondrial DNA), SNP (single nucleotide polymorphism) and SSR (simple sequence repeat) [11]–[13]. Microsatellites are widely used as genetic markers in the studies of marine mammals [14], [15]. Owing to limited genomic sequences, only few microsatellites have been successfully developed in cetaceans. The availability of abundant genomic sequence information of the Indo-Pacific humpback dolphin would be benefit to the development of more genetic markers, as well as the investigations of the underlying molecular mechanism of immune response and adaptive evolution in cetaceans.

Genome sequencing and global exploration of transcriptome are effective methods to obtain abundant functional sequences involved in various biological processes. Compared to the whole-genome sequencing, the next-generation RNA sequencing technologies provide a cost-effective approach to produce sequences of the transcribed portion of genes. Several transcriptome studies indicated that it was feasible for plant and animal species to assemble and analyze the transcriptome with Illumina second generation sequencing technology [16]–[18]. In this study, we performed the analyses of the leucocyte transcriptome of the Indo-Pacific humpback dolphin, including transcriptome sequencing, assembly and annotation. A large number of genes involved in the immune response and adaptive evolution of cetaceans were identified. This transcriptome dataset provided the first picture of the genomic transcriptional activity of this endangered marine mammal species, and moreover, a valuable resource for identification of genes involved in immune response and adaptive evolution, identification of new genes as well as for development of genetic markers in the Indo-Pacific humpback dolphin.

## Results and Discussion

### Illumina Sequencing and Sequence Assembly

**Figure 1:**
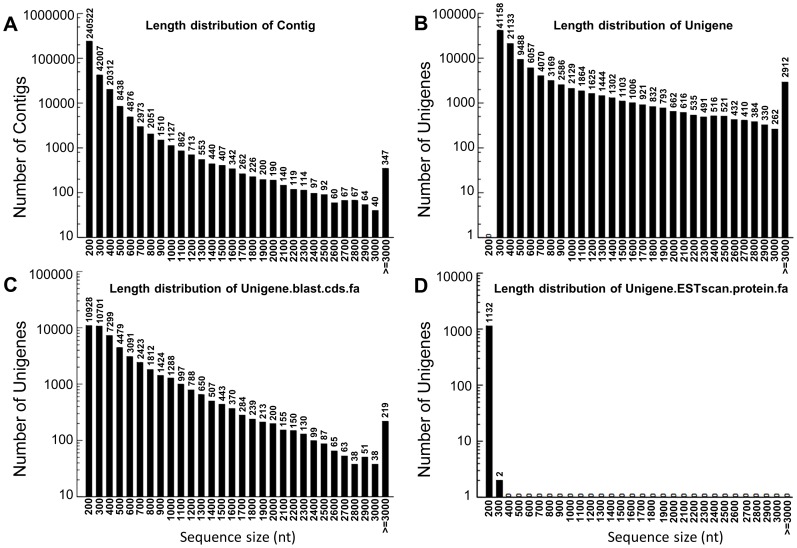
Overview of the Indo-Pacific humpback dolphin leucocytes transcriptome assembly. (A) The size distribution of the contigs obtained from *de novo* assembly of high-quality clean reads. (B) The size distribution of the unigenes produced from further assembly of contigs. (C) The size distribution of the CDS produced by searching unigene sequences against various protein databases (NR, Swiss-Prot, KEGG and COG, in order) using BLASTX (E-value<10^−5^). (D) Size distributions of the ESTs obtained from the ESTScan results. For unigene CDS that had no hits in the databases (NR, Swiss-Prot, KEGG and COG), the BLAST results were subjected to ESTScans and then converted into peptide sequences.

In total, illumina sequencing yielded 52,178,320 reads from the mRNA pool of the leucocytes of the Indo-Pacific humpback dolphin. After removal of adaptor sequences, ambiguous reads and low-quality reads (Q20<20), we obtained 47,840,388 clean reads comprising 4,305,634,920 nucleotides. The Q20 percentage (sequencing error rate <1%) and GC percentage are 97.58% and 51.55%, respectively. All clean reads were assembled *de novo* using the Trinity program [19] as summarized in Table 1. The 47,840,388 clean reads were further assembled into 329,213 contigs with a mean length of 209 bp and an N50 of 245 bp (i.e. 50% of the assembled bases were incorporated into contigs of 245 bp or longer). The length distribution for all contigs was presented in Figure 1A. Although most of the contigs (240,522) were less than 200 bp, 17,934 contigs were longer than 500 bp. From the contigs, 108,751 unigenes comprising 81,347 singletons and 27,404 clusters were obtained with an average unigene length of 671 bp and an N50 of 1,114 bp (Table 1). Singletons represent the reads with similarities to other reads, but with minor differences resulting in the exclusion from the clusters. Figure 1B showed the length distributions of all assembled unigenes. 7,409 of the 108,751 unigenes were longer than 2,000 bp.

**Table 1:** Summary of the sequence assembly after Illumina sequencing.

	Total Number	Total Length(nt)	Mean Length(bp)	N50(bp)	Distinct Singletons	Distinct Clusters
Raw sequencing reads	52,178,320	47,840,388				
Total Clean reads	47,840,388	4,305,634,920				
Total Contigs	329,213	68,847,299	209	245		
Total unigenes	108,751	72,951,473	671	1114	81,347	27,404
GC percentage				51.55%		
Q20 percentage				97.58%		
N percentage				0.00%		

For coding sequence (CDS) analysis, protein prediction and gene annotation, all assembled unigenes were searched against various databases: GenBank non-redundant (NR), Swiss-Prot, Gene Ontology (GO), Clusters of Orthologous Groups (COG) [20], and Kyoto Encyclopedia of Genes and Genomes (KEGG) using the BLASTx program (E-value<10^−5^). In total, 49,221 significant BLAST hits (45.26% of all unigenes) were obtained. The CDS of the unigenes that did not have BLAST hit were converted into deductive peptide sequences using ESTScan [21]. The length distributions for all the CDS were shown in Figure 1C and 1D.

### Functional Annotation

**Figure 2:**
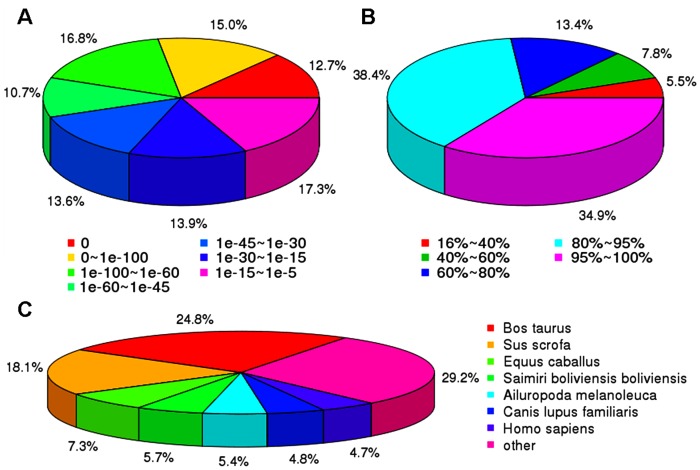
Characterization of the assembled unigenes against NR protein databases. (**A**) E-value distribution of BLAST hits for the assembled unigenes with a cutoff of 1E-5. (**B**) Similarity distribution of the top BLAST hits for the assembled unigenes with a cutoff of 1E-5. (**C**) Species distribution of the top BLAST hits for the assembled unigenes with a cutoff of 1E-5.

For validation and annotation of the assembled unigenes, all the assembled unigenes were searched against the NR, Swiss-Prot protein databases and NCBI nucleotide sequences database (NT) using BLASTx program (E-value<10^−5^). The results showed that 48,868 and 46,587 unigene sequences had BLAST hits to annotated proteins in NR and Swiss-Prot protein databases, respectively (Table 2). Analysis of the distributions of E-values indicated that 82.7% of the aligned sequences showed significant homologies to the entries in the NR database (E-value<10^−15^) (Fig. 2A). Further analysis of the similarity distributions indicated that 73.3% of matched sequences had alignment identities greater than 80% (Fig. 2B). A large part of the hits matched the sequences of *Bos Taurus* (24.8%), *susscrofa* (18.1%), and the others were identified within the reference protein databases of *Equuscaballus* (7.3%), *Saimiriboliviensis* (5.7%), *Ailuropodamelanoleuca* (5.4%), *Canis lupus familiaris* (4.8%), and *Homo spapiens* (4.7%), respectively (Fig. 2C). There were also many unigenes without any BLAST hit, which might represent additional genes that had not represented in the annotated protein databases or sequences that were too short to produce hits. In addition, BLASTx of the assembled unigene sequences against NT database resulted in the identification of 83,676 sequences with at least one significant alignment to an existing gene model (Table 2).

**Table 2:** Summary of the unigene hits in public protein databases.

Public proteindatabase	Number of unigene hits	Percentage (%)
NR	48,868	57.68
Swiss-Prot	46,587	54.99
KEGG	36,345	42.90
COG	16,467	29.44
GO	37,976	44.83
NT	83,676	98.77
ALL	84,716	

**Figure 3:**
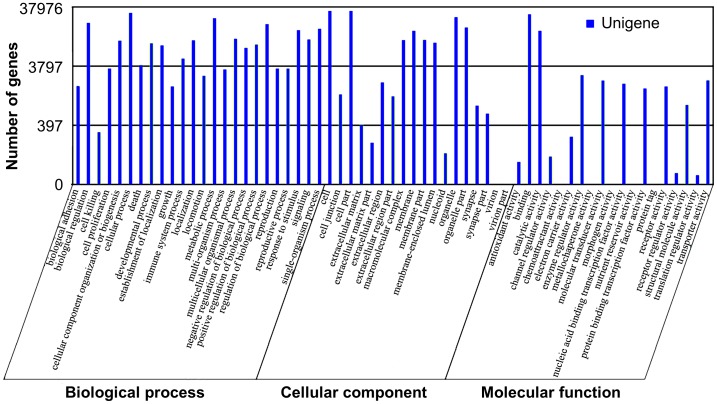
GO assignment for assembled unigenes. The results are summarized in three main categories: biological process, cellular component and molecular function. In total, 37,976 unigenes were assigned to GO. Classified gene objects are depicted as absolute numbers of the total number of gene objects with GO assignments.

GO (gene ontology) is an international classification system for standardized gene functions and is used to annotate and analyze gene functions and gene products in any organism. GO contains three main, independent ontologies: biological process, molecular function, and cellular component [17]. To predict their possible functions, the unigenes were searched against the GO database. We used the Blast2GO program [22] to analyze GO annotation of the assembled unigenes, and then applied the WEGO software to perform GO functional classifications [23]. Based on NR annotation, 37,976 unigenes were assigned to 61 GO terms belonging to three main GO ontologies (Fig. 3). Further analysis of the 61 GO terms showed that the dominant terms were “cellular processes”, “metabolic processes”, “cells”, “cell parts”, “organelles” and “binding”. Within the biological process group, the great majority was related to cellular process and metabolic process. Within cellular component, the largest proportion was assigned to cells and cell parts, followed by binding and catalytic activity. Remarkably, a few genes were related to immune system process and locomotion.

**Figure 4:**
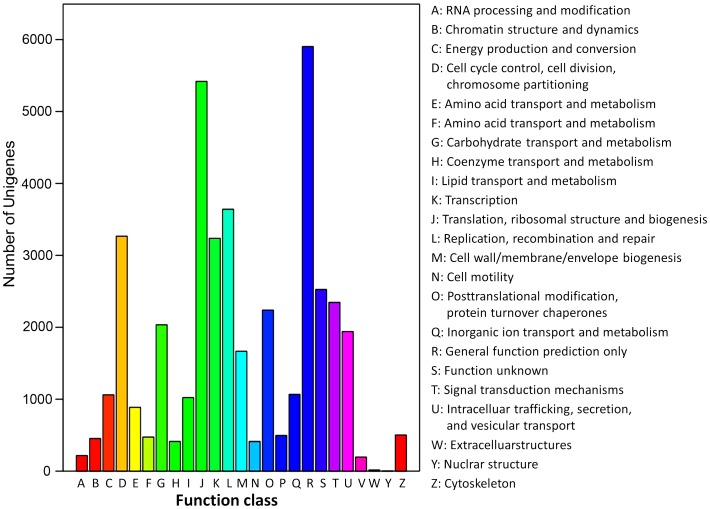
Clusters of orthologous group (COG) classification. In total, 16,467 of the 48,868 sequences with NR hits were grouped into 25 COG classifications.

The COG database represents an attempt on a phylogenetic classification of the proteins encoded in complete genomes, and is applied to the function prediction and classification of new sequences [16]. In order to predict their possible functions, the unigenes were searched against the COG database. The result showed that 16,467 unigenes of 48,868 NR hits were clustered into 25 functional categories among which “general function prediction only” represented the largest group (5,903 unigenes, 35.85%), followed by “translation, ribosomal structure and biogenesis” (5,419 unigenes, 32.91%), “replication, recombination and repair” (3,641 unigenes, 22.11%) and “cell cycle control, cell division, chromosome partitioning” (3,267 unigenes, 19.84%). The smallest groups were “nuclear structures” (4 unigenes) and “extracellular structures” (16 unigenes) (Fig. 4).

KEGG, a pathway-based categorization of orthologous genes, provides useful information for predicting the functional profiles of genes [24]. To identify the biological pathways that are active in leucocytes of the Indo-Pacific humpback dolphin, all the annotated sequences were mapped to the reference canonical pathways in KEGG. In total, 36,345 unigenes were grouped into 258 KEGG pathways (Table S1). Leucocytes include many cells of the immune system involved in defending the body against both infectious disease and foreign materials, so it is unsurprising that a larger number of unigenes were mapped to pathways involved in immune response, such as chemokine signaling pathway (1,127 unigenes), T cell receptor signaling pathway (746 unigenes), B cell receptor signaling pathway (668 unigenes), toll-like receptor signaling pathway, (529 unigenes), cytokine-cytokine receptor interaction (514 unigenes), RIG-I-like receptor signaling pathway (373 unigenes), NOD-like receptor signaling pathway (362 unigenes), antigen processing and presentation (314 unigenes). In addition, we also identified some genes involved in the pathways related to the adaptive evolution of cetaceans, such as Fat digestion and absorption (118 unigenes), Vasopressin-regulated water reabsorption (114 unigenes), Glyoxylate and dicarboxylate metabolism (112 unigenes), Fatty acid elongation (53 unigenes), Fatty acid biosynthesis (48 unigenes), Renin-angiotensin system (21 unigenes). These genes might be related to fat storage, energy metabolism, and osmoregulation in cetaceans.

### SSR and SNP Markers Identification

Microsatellites, also known as SSRs, are classes of repetitive DNA sequences which are ubiquitous in eukaryotic genomes [25]. It is well-known that SSRs are ideal markers for paternity determination, population genetics investigation, genetic diversity assessment and genetic map development [26]. From the 108,751 assembled unique sequences, 9,906 SSRs (9,457 with simple repeats and 449 with compound formation) were identified in 8,762 unique sequences, in which 950 sequences contained more than one SSR. Monomer repeats (43.33%) were the most common SSRs, followed by di- (29.50%), tri- (20.54%), tetra- (3.06%), hexa- (2.33%), and pentanucleotide (1.24%) repeats. All the SSRs were further classified by the number of repeat units. The results showed that the number of potential SSR composed of 6 repeat units was the most (16.62%, 1,646), followed by 5 (14.84%, 1,470), 12 (10.47%, 1,037) and 7 repeat units (9.39%, 930). Additionally, 4,306 potential SSRs contained more than 12 repeat units, and the motifs almost were mono-nucleotide repeats (Table S2). In the assembly, the most frequent SSR motifs were mono-nucleotide A/T (41.59%), followed by AC/GT (19.46%), AG/CT (6.7%), and AGC/CTG (5.18%).

In addition, 3,681 putative SNPs containing 2,591 (70.39%) transitions (Ts) and 1,090 (29.61%) transversions (Tv) were identified in the assembled sequences (Table S3). The frequencies of different Ts types were similar, and those of Tv types were a little of difference. The transcriptome wide Ts/Tv ratio was 2.37 (Table S3).

The SSRs and putative SNPs in the leucocyte transcriptome of the Indo-Pacific humpback dolphin would provide potential genetic markers for the applications in population genetics, comparative genomics, as well as gene-based association studies aimed to understanding the genetic control of adaptive traits.

### Identification of Sequences Related to the Immune Response

Leucocytes play an important role in the defense system which resists and destroys pathogenic microorganisms by phagocytosis and generation of antibodies. A key word list and GO immune-related terms were used to search for genes putatively involved in the immune system of the Indo-Pacific humpback dolphin. We obtained a large number of immune-related genes which were involved in common, well-recognized immune pathways, such as antigen processing and presentation, cell recognition, complement and coagulation cascades, toll-like receptors (TLRs), T cell receptor signaling, cell receptor signaling and so on (Table S4). The toll receptor, as the signal transducer of the Toll pathway, plays a crucial role in innate immune response. In this study, we identified ten genes coding toll receptors in transcriptome datasets, including TLR1 to TLR10. Furthermore, we discovered a few genes belonging to the TLR signaling pathway, such as MyD88 and mitogen-activated protein kinases (MAPKs). JAK (Janus kinase) is a family of intracellular, non-receptor tyrosine kinases that transduces cytokine-mediated signals via the JAK-STAT pathway [27]. Many studies have shown that signal transducers and activators of transcription proteins (STAT) are involved in the development and function of the immune system and play a role in maintaining immune tolerance and tumor surveillance [28]. In our study, we identified abundant unigenes with high similarity to all the seven mammalian STAT family members (STAT1, STAT2, STAT3, STAT4, STAT5A/5B, and STAT6) and the members of the Janus family (JAK1, JAK2, JAK3 and tyrosine kinase 2). The identification of JAK-STAT pathway-related genes will be useful for learning more about the complexities of immune responses in the Indo-Pacific humpback dolphin. In addition, signaling and interaction molecules were evidenced in the transcriptome such as cytokines and cytokine receptors. Besides, proteases, protease inhibitors and stress proteins (such as heat shock proteins and metallothionein) were also found in our dataset.

Further analysis of the immune-related genes indicated that most of them were involved in the innate immune response. It is explicit that environmental contaminants and microparasites, including viruses, bacteria and protozoans, may constrain the growth of wild animal populations [29]. To date, many microparasites have been detected in marine animals, including *Paramyxoviridae, Poxviridae, HerpesvIridae, Adenoviridae, and Caliciviridae* [30], [31]. These microparasites interfered with population abundance by inducing high mortalities, lowering reproductive success or synergistically increasing the virulence of other diseases. In the past decades, reports on the mechanisms of immune response to microparasites infection in cetaceans were rare. The knowledge about the cetacean immune system is still fragmentary and several aspects of immunomodulatory xenobiotics are under debate [9], [32]. Innate immunity is the first line of host defense against pathogens. Many immune cells, such as monocytes, macro-phages, leukocytes (PMN) and NK cells, are involved in the detection and removal of microbial pathogens [33]. Compared to those from other mammals, few immune related genes of the Indo-Pacific humpback dolphin have been identified. Our results revealed a large number of innate immune-related genes, covering almost all known innate immune pathways, such as pathogen recognition, modulation and signaling, which would facilitate our comprehensive understanding of the mechanisms involved in the immune response to microparasites infection in cetaceans. In addition, it is known that immune system genes undergo more adaptive evolution than non-immune system genes [34], [35]. TLR4 gene had been used to reveal the evolutionary history of pattern recognition molecules across cetaceans and their closest terrestrial relatives [36]. The immune-related genes of the Indo-Pacific humpback dolphin would provide an abundant resource for understanding of cetacean evolution and their adaptation to the aquatic environment.

### Identification of Sequences Related to Adaptive Evolution and Cetacean-specific Traits

To adapt the transition from land to aquatic environment, cetaceans had gradually formed some tremendous changes in morphology and physiology. A series of changes must have accordingly occurred at molecular level to allow the necessary morphological and physiological adaptations. Our results showed that there were a number of functional categories that might be correlated with the adaptive evolution and cetacean-specific traits which might be related to fat storage, echolocation, energy metabolism, osmoregulation and locomotion (Table S5). Meanwhile, a large number of genes showing significant enrichment in these functional categories were identified (Table S6). Many cetaceans have a thickened fat layer called blubber, which acts as their primary location for fat storage. It was reasonable that a few of genes related with fat storage were detected. To adapt the higher energy cost during locomotion underwater, the Indo-Pacific humpback dolphin had many mitochondrion-associated genes. Many mitochondrion-associated genes were found in the transcriptome. A few of genes related to the response to osmotic stress, renin-angiotensin system, urea transport or hyperosmotic response were also identified. In addition, there were also some genes that were particular for cetacean-specific traits, such as echolocation. Among of these genes, chromodomain helicase DNA binding protein 7, Solute carrier family 12 member 7, transcription factor 25, and ADAM metallopeptidase domain 19 are closely associated with hearing. Many cetaceans have nasal structures that generate echolocation signals, enabling them to use sound to locate prey or navigate past obstacles [37]. Nasal embryonic luteinizing hormone-releasing hormone factor and vomeronasal 1 receptor, which function in the nasal development, were detected in the transcriptome. Furthermore, we identified many genes putatively related to other cetacean-traits, including genes that were involved in cardiovascular system development (PLA2G5, disintegrin and metalloproteinase domain-containing protein 15), nervous system development (SMARCB1, formin-binding protein 1) and sperm function and spermatogenesis (nanos1, spermatogenesis associated 7).

Analysis of adaptive evolution at the molecular level achieves great insights into the mechanisms underlying the evolution of complex phenotypes. Genomic sequencing contributes to clarifying the influence of natural selection on an organism’s evolutionary history [38]. As a unique clade of mammalian, cetaceans have developed various strategies morphologically, physiologically and ecologically in order to adapt to their aquatic environments [10], [39]. The molecular mechanisms underlying these adaptations are still poorly understood. Our results showed that there were large numbers of genes involved in the adaptation of the Indo-Pacific humpback dolphin to the aquatic environment. These genes are significantly enriched in the categories of lipid transport, glycolysis, ATPase activity, aerobic metabolism, sense perception of sound, osmoregulation and muscle organ development. Most cetaceans inhabit the hyperosmotic marine environment, but a few species live in the hypoosmotic freshwater. However, despite the obvious differences of their living environment, both freshwater and marine cetaceans face the same challenges of body balance and electrolyte homeostasis in water. Some reports suggested that the osmoregulation in cetaceans was relevant to the metabolism of water/electrolytes, morphology and histology of the kidney and skin, hormone regulation, as well as the specific molecules [40]–[42]. Malvin and Vander reported that there was a renin-angiotensin system in the cetaceans, which might play an important role in the electrolyte balance of aquatic mammals, particularly for Na^+^ reabsorption [43], [44]. The identification of genes involved in the renin-angiotensin system in the leucocyte transcriptome further confirmed the existence of renin-angiotensin system in cetaceans. Aquaporins (AQPs) are intrinsic membrane proteins and play an important role in water channels of many cell types [45]. There are thirteen known types of AQPs in mammals; six of them are located in the kidney [46]. Recently, AQP2 and AQP1 had been reported to localize to the kidney in the cetaceans [47], [48]. In our study, the AQP7 gene was detected in the transcriptome. AQP7 facilitates water, glycerol and urea transport and plays a crucial role in metalloid homeostasis [45]. Further investigations are needed to know its role in the regulation of water metabolism in cetaceans.

## Conclusions

In this study, we characterized the leucocyte transcriptome of the Indo-Pacific humpback, and identified thousands of genetic markers (SSRs and SNPs) and abundant specific gene families involved in immune response and adaptive evolution. This is the first investigation on the whole transcriptome of this endangered species. The dataset provides a substantial genomic-level resource for the endangered species and will be useful in understanding of the molecular mechanisms of various pathways in cetaceans, including immune response and adaptive evolution.

## Materials and Methods

### Blood Sampling, Leukocyte Isolation and RNA Extraction

Work with the Indo-Pacific humpback dolphin in this study was specifically approved by the Ministry of Agriculture of China under permit number 2012–31. The protocol was specifically proved by the Administration of Ocean and Fisheries of Guangdong Province, China under permit number 2012–647. No issue on ethics was concerned in this study. The operation of blood sampling was carried out by veterinarians with professional training. The blood sample of the Indo-Pacific humpback dolphin was obtained from a wild male adult individual that was rescued for rehabilitation from a recent animal live-stranding event in a shallow river near Foshan city of China. The sample site on the tail fin was sterilized with surgical cotton containing 70% alcohol, and 6 ml of blood was taken with a sterile syringe from the vein of the tail fin. The fresh blood was collected into EDTA-containing tubes, and then centrifuged for 5 min at 400–500 g. The supernatant was discarded and 18 ml of Red Blood Cell Lysis Buffer (Beyotime Institute of Biotechnology) were added, mixed gently by flicking the tubes and lasted for 10 min. The tubes were then centrifuged at 1000 g for 5 minutes at 4°C to collect the leukocytes.

Total RNA was extracted from the leucocytes of Indo-Pacific humpback dolphin using TRIzol reagent (Invitrogen) according to the manufacturer’s protocol. Total RNA was treated with RNase-free DNase I (Promega) for 30 min at 37°C to remove residual DNA. RNA purification was carried out using RNeasy Mini Kit (Qiagen) following the manufacturer’s instructions.

### cDNA Library Construction and Sequencing

Illumina sequencing was performed at Beijing Genomics Institute (BGI)-Shenzhen, China. mRNA with poly(A) tail was isolated from 20 µg total RNA treated with DNase I using Magnetic Oligo (dT) Beads (Illumina). The mRNA was fragmented into small pieces (200–700 bp) by treatment with divalent cations at 94°C for 5 minutes. With random hex-amer primers (Illumina), the double-stranded cDNA was synthesized using the SuperScript double-stranded cDNA synthesis kit (Invitrogen) and was further subjected to end-repair using T4 DNA polymerase, the Klenow fragment, and T4 polynucleotide kinase followed by a single A base addition using Klenow 3′ to 5′ exo-polymerase, then was ligated with an adapter or index adapter using T4 DNA ligase. To select the proper templates for downstream enrichment, the products of ligation reaction were purified on 2% agarose gel. The cDNA fragments (about 200 bp) were recovered from the gel. Fifteen rounds of PCR amplification were carried out to enrich the purified cDNA template using PCR primer PE 1.0 and 2.0 (Illumina) with Phusion DNA polymerase. Finally, the cDNA library was constructed with 200 bp insertion fragments. After validating on an Agilent Technologies 2100 Bioanalyzer, the library was sequenced using Illumina HiSeq™ 2000 according to the manufacturer’s instruction.

### Data Filtering and *de novo* Assembly

Before the transcriptome assembly, we carried out a stringent filtering process of raw sequencing reads. The raw reads were cleaned by removing adapter sequences, non-coding RNA (such as rRNA, tRNA and miRNA), low-quality sequences (reads with ambiguous bases ‘N’), and reads with average length less than 20 bases. *De novo* transcriptome assembly was performed by Trinity program as described elsewhere [19]. Briefly, Trinity first combines reads of a certain length of overlap to form longer fragments without N (gaps), which are called contigs. These contigs will be further processed for sequence clusters with the sequence clustering software TGICL [49], and these sequences are defined as unigenes. The calculation of unigene expression used the RPKM method [50], which was able to eliminate the influence of different gene lengths and sequencing discrepancy on the calculation of gene expression. The sequence dataset generated in this study is available at the European Nucleotide Archive (http://www.ebi.ac.uk/ena/) under the accession number ERP003522.

### Annotation and Classification of Unigenes

All unigenes were employed for homology search against various protein databases in following order: NR, Swiss-Prot, KEGG and COG with BLAST program (E-value<10^−5^), and the best aligning results were selected to annotate the unigenes. If the aligning results from different databases are in conflict with each other, the results from NR database were preferentially selected, followed by Swiss-Prot, KEGG and COG database. The DNA sequences obtained from the BLAST searches were then used to extract CDS from the unigene sequences, and were then converted into peptide sequences. For unigenes that did not align to any of the above databases, ESTScan software [21] was used to predict their coding regions and decide sequence direction.

To further annotate the unigenes, the Blast2GO program was used to get GO annotation [22]. The WEGO software was then used to perform GO functional classification of all unigenes to view the distribution of gene functions of the species at the macro level [23]. The unigene sequences were also aligned to the COG database to predict and classify possible functions. Pathway assignments were performed according to KEGG pathway database [24].

### SSR and SNP Markers Identification

MicroSAtellite (MISA) was used to identify microsatellites in the unigenes. The parameters were adjusted in order to identify perfect mono-, di-, tri-, tetra-, penta-, and hexanucleotide motifs with a minimum of 10, 6, 5, 5, 4, and 4 repeats, respectively. Unique sequences containing 150-bp sequence on both sides of the microsatellite repeat were considered sufficient for primer design [51].

For putative SNP identification, SOAPsnp was used to screen in the unigenes (http://soap.genomics.org.cn/soapsnp.html) [52]–[54].

### Identification of Immune-Related Genes

The identification of immune-related genes was performed as described by Pereiro *et al*. [55] with some modifications. GO terms at level 2, 3 and 4 directly related to immunity were used for selecting putative immune-related genes. All the genes were further analyzed based on an extensive list of immune terms and a comprehensive literature review (Table S7). In order to find more genes belonging to the relevant immune-pathways in the transcriptome sequences, we used the KEGG reference pathways as a template for constructing the following immune-cascades: Complement pathway, Toll-like receptor signaling pathway, B cell receptor signaling pathway, Chemokine signaling pathway, Lysosome, Jak-STAT signaling pathway, RIG-I-like receptor signaling pathway, NOD-like receptor signaling pathway, T cell receptor signaling pathway and apoptosis cascade. Additional molecules were included in some cases after bibliographic review.

### Identification of Adaptive Evolution and Cetacean-specific Traits Genes

GO terms at level 2, 3 and 4 directly related to adaptive evolution and cetacean-specific traits were used for selecting putative adaptive evolution and cetacean-specific traits-related genes. The following features were selected to detect relevant GO terms: fat storage, echolocation, energy metabolism, osmoregulation and locomotion.

## Supporting Information

Table S1 **Pathway enrichment analysis for the leucocyte transcriptome of the Indo-Pacific humpback dolphin.**
(XLS)

Table S2 **Number of SSRs detected in the leucocyte transcriptome of the Indo-Pacific humpback dolphin.**
(XLS)

Table S3 **Number of each type of SNPs detected in the leucocyte transcriptome of the Indo-Pacific humpback dolphin.**
(XLS)

Table S4 **Putative sequences related to the immune response.**
(XLS)

Table S5 **Some functional categories correlated with the adaptive evolution and cetacean-specific traits.**
(XLS)

Table S6 **Putative sequences related to adaptive evolution and cetacean-specific traits.**
(XLS)

Table S7 **GO terms and literatures used for searching for the immune-related genes.**
(XLS)
